# Theoretical Investigation of Hydrogen Production from Alkaline Media Through TiO_2_-Supported Triple-Atom Catalysts

**DOI:** 10.3390/ma19112217

**Published:** 2026-05-25

**Authors:** Guangce Zhao, Gang Zhou

**Affiliations:** School of Science, Hubei University of Technology, Wuhan 430068, China

**Keywords:** triple-atom catalyst, Ni_3_/TiO_2_, alkaline hydrogen evolution reaction, density functional theory, delocalized electronic states, implicit solvation model

## Abstract

Developing low-cost, non-noble-metal electrocatalysts to replace platinum-based benchmarks for the alkaline hydrogen evolution reaction (HER) remains a critical challenge. Using density functional theory (DFT) calculations combined with the computational hydrogen electrode (CHE) model, we systematically investigate the thermodynamics, kinetics, and intrinsic reaction mechanism of HER on a TiO_2_-supported Ni_3_ trimer (Ni_3_/TiO_2_) in alkaline media. We find that the Ni_3_ trimer, rather than the TiO_2_ support, provides multiple active sites for intermediate adsorption. The trimeric Ni_3_ motif generates delocalized electronic states, leading to electron-rich active sites that significantly lower the barrier for water dissociation, facilitate intermediate desorption, and sustain catalytic turnover. The reaction proceeds predominantly via the Volmer–Heyrovsky pathway, where either water dissociation or H_2_ desorption can be the rate-determining step, depending on the applied potential. Crucially, the significantly reduced reaction barrier heights demonstrate that the alkaline HER activity of Ni_3_/TiO_2_ is comparable to that of benchmark Pt_1_/TiO_2_ single-atom catalysts (SACs). This work establishes a promising design strategy for constructing high-performance non-noble metal few-atom catalysts (FACs) to replace noble metal SACs for multi-step electrocatalytic reactions.

## 1. Introduction

Within the increasingly promising family of few-atom catalysts (FACs) [1,2,3,4], triple-atom catalysts (TACs) have garnered extensive research attention in recent years. Their widely reported superior performance stems from two core attributes. First, the three metal atoms in supported transition-metal (TM) trimers adopt a triangular configuration that serves as the crystal embryo of the bulk parent phase [5]—that is, the minimum structural unit—regardless of whether the bulk metal adopts a face-centered cubic (FCC) or body-centered cubic (BCC) lattice structure. Second, these trimeric structures feature abundant, versatile active sites. During distinct reactions, or even across different stages of a single reaction, active sites including isolated metal centers, edge bridge sites, and hollow interstitial sites within the three-metal motif can be sequentially activated, thereby participating in the reaction either independently or synergistically [6]. While this unique property markedly boosts the thermodynamic and kinetic feasibility of multi-step reactions, it simultaneously renders the corresponding reaction mechanisms and pathways far more intricate and challenging to elucidate.

The earliest seminal study in this field was reported by Socaciu et al. [7], who documented an exceptionally high turnover frequency (TOF) for Ag_3_ clusters in the low-temperature catalytic epoxidation of propylene, with performance even surpassing that of gold clusters. Subsequent density functional theory (DFT) calculations revealed that the protruding triangular active sites of Au_3_ trimers were responsible for their high activity toward CO oxidation [8]. In this size-constrained system with a limited number of metal atoms, co-adsorbed CO molecules at the active sites facilitate O–O bond scission during CO oxidation, yielding two CO_2_ molecules. Over the past two years, synthetically fabricated C,N,S-doped Fe(Al)OOH-NF metal (oxy)hydroxide TACs have been shown to exhibit excellent catalytic activity and durability for overall water splitting [9]. Shortly thereafter, asymmetric Pt–Ru–Co TACs were fabricated via selective atomic layer deposition, and experimental results verified that these catalysts deliver outstanding electrocatalytic performance for both the hydrogen evolution reaction (HER) and hydrogen oxidation reaction (HOR), outperforming their single-atom catalyst (SAC) and dual-atom catalyst (DAC) counterparts. This performance enhancement is attributed to the interfacial interactions between Pt/Ru and Co moieties, which effectively accelerate reaction kinetics [10].

Collectively, these pioneering studies have firmly established the unique advantages of TACs over lower-nuclearity SACs and DACs in a wide range of catalytic reactions. Compelling evidence to date demonstrates that, benefiting from their abundant multi-active sites, TACs outperform SACs for complex multi-step reactions involving multi-intermediate transformations, multi-atom metallic configurations, and surface sites with metal–metal bonds [9,10]. Unlike SACs that only provide isolated single-metal sites, the triangular trimer motif of TACs enables sequential activation of top, bridge, and hollow sites across different elementary steps, allowing precise regulation of the adsorption and desorption behaviors of multiple reaction intermediates. Nevertheless, systematic investigations into the structure–activity relationships and interfacial synergistic effects of TACs remain surprisingly limited. In particular, the confinement effect of delocalized metallic states among transition metal (TM) atoms, which is induced by the coordination environment of the supporting substrate [5], along with the distinct role of these delocalized states in multi-step catalytic processes, has rarely been explored in a systematic manner. This fundamental issue is critical for elucidating the atomic size-dependent confinement effect and metal–support synergistic interactions of FACs, as well as their underlying impacts on catalytic performance and multi-step reaction mechanisms.

The acidic HER, which involves only one-electron transfer, enables direct and intuitive interrogation of the active sites and intrinsic catalytic properties of supported FACs, as well as elucidation of their preferred reaction pathways. Indeed, our earlier work on the atomic size-dependent confinement and metal-support synergism in Ni*_n_*/TiO_2_ (*n* = 1–3) FACs for acidic HER revealed that Ni-SAC exhibits 100% atomic utilization but the lowest catalytic activity; Ni-DAC retains 100% atomic utilization while delivering the highest catalytic activity; and Ni-TAC shows the lowest atomic utilization (1/3) with only moderate catalytic performance [3]. These findings provide a fundamental understanding of the atomic size effect in FACs for simple proton reduction reactions, but they cannot be directly generalized to more complex reaction systems that require multiple elementary steps. In stark contrast, the alkaline HER—of greater technological relevance to industrial alkaline water electrolysis—involves a more complex multi-step reaction process with an additional rate-limiting water dissociation step prior to hydrogen formation, placing far more stringent demands on the design of catalysts with multifunctional active sites. This makes TACs with versatile active sites and tunable delocalized electronic structures particularly promising for alkaline HER, yet the underlying reaction mechanism, electronic origin of activity, and structure–activity relationships remain poorly understood.

Recently, motivated by the surging global demand for hydrogen energy and the rapid development of the hydrogen economy [11,12,13], Vega et al. computationally predicted six ternary TM1-TM2-Ni co-doped TAC alloy systems via density functional theory (DFT) calculations, which exhibit robust structural stability, moderate hydrogen adsorption free energy, and excellent anti-coking performance, making them suitable for hydrogen production integrated with bio-oil upgrading [14]. Meanwhile, Ru_3_ trimers dispersed on oxidized carbon nanotubes have been synthesized and demonstrated to deliver exceptional catalytic performance for the alkaline HER: the overpotential in 1 M KOH solution outperforms commercial 20 wt.% Pt/C and 5 wt.% Ru/C; the mass activity is 23.47 and 11.83 times higher than that of commercial Pt/C and Ru/C, respectively [15]. Among various oxide-supported non-noble metal catalysts, TiO_2_-supported Ni-based catalysts have attracted extensive attention, and Ni/TiO_2_ has emerged as a cost-effective alternative to noble metal-modified TiO_2_ photocatalysts for solar-driven hydrogen evolution in alkaline media [16]. However, the alkaline HER mechanism of TiO_2_-supported Ni_3_ TACs, as well as the intrinsic correlation between the delocalized electronic states of the Ni_3_ trimer and its catalytic performance, has not been systematically elucidated to date.

Addressing this critical knowledge gap requires a comprehensive theoretical investigation that can unravel the atomic-level details of the catalytic process and establish clear structure-activity relationships. Inspired by these advances, we herein performed a systematic DFT investigation of the alkaline HER over a TiO_2_-supported Ni_3_ TAC (Ni_3_/TiO_2_). The catalytic activity and underlying reaction mechanism of Ni_3_/TiO_2_ were comprehensively explored via Gibbs free energy calculations and climbing-image nudged elastic band (CI-NEB) simulations. We further analyzed the intrinsic electronic origin of the energy barrier for each elementary step and elucidated the unique regulatory role of the Ni_3_/TiO_2_ interface throughout the entire catalytic cycle. This work aims to uncover the inherent correlation between catalytic performance and the structural distortion, as well as the electronic state evolution of Ni_3_/TiO_2_ along the reaction pathways. Finally, we evaluated the practical application potential of the Ni_3_/TiO_2_ TAC for alkaline hydrogen production, with the goal of providing a promising low-cost, high-performance non-noble metal alternative to TiO_2_-supported noble-metal SACs for alkaline HER.

## 2. Methodology and Calculation Model

All spin-polarized DFT calculations were performed using the Vienna Ab initio Simulation Package (VASP) [17]. The projector augmented wave (PAW) method [18] was employed to describe electron-core interactions, with a plane-wave basis set expanded to a kinetic energy cutoff of 500 eV. The Perdew–Burke–Brinkerhoff (PBE) functional within the generalized gradient approximation (GGA) was adopted to describe the exchange-correlation potential [19]. For the TiO_2_ substrate, the DFT + U approach [20] was applied to account for the on-site Coulombic interactions of localized d/f orbitals in transition metal and rare-earth compounds via an additional Hubbard-type effective parameter (U*_eff_*). An effective Hubbard parameter of U*_eff_* = 7 eV was set for the Ti 3d orbitals, which has been extensively validated in prior literature to accurately reproduce the band structure and lattice parameters of anatase TiO_2_ [21,22]. Specifically, the bulk anatase TiO_2_ unit cell was optimized using an 11 × 11 × 3 Monkhorst–Pack k-point mesh [23]. The optimized lattice parameters (a = b = 3.803 Å, c = 9.748 Å) are in excellent agreement with experimental values [24]. The calculated band gap is 3.15 eV, which is very close to the experimental result of 3.20 eV [25]. Convergence tests confirmed that increasing the plane-wave cutoff energy to 600 eV only changes the total energy by 0.008 eV, with negligible variation in the lattice constant.

The Ni_3_/TiO_2_ (101) model was constructed based on well-established theoretical protocols [3,5], which have demonstrated that triangular Ni_3_ trimers can be stably anchored on metal oxide surfaces. Owing to the coordination effect of unsaturated O and Ti atoms on the TiO_2_ (101) surface, the triangular configuration of the Ni_3_ trimer is more stable than its linear-chain configuration along the <100> direction, with a total energy difference of approximately 0.3 eV between the two structures [5]. A three-layer Ti_2_O_4_ slab, where each layer corresponds to a stoichiometric Ti_2_O_4_ unit, was cleaved from the fully optimized anatase TiO_2_ bulk structure (lattice constants pre-optimized with U*_eff_* = 7 eV) to model the thermodynamically most stable (101) facet. A 2 × 2 supercell was employed, with a vacuum spacing of no less than 20 Å between adjacent slabs to eliminate spurious inter-slab interactions. Brillouin zone sampling was performed using a 3 × 3 × 1 Monkhorst–Pack k-point mesh. Dipole corrections were applied to account for the asymmetric charge distribution across the slab. During geometry optimization, the top two Ti_2_O_4_ layers, the anchored Ni_3_ trimer, and all adsorbed species were fully relaxed, while the bottom Ti_2_O_4_ layer was fixed at its optimized bulk-truncated geometry to simulate the semi-infinite bulk substrate. The convergence thresholds for the electronic self-consistent field (SCF) iterations and ionic relaxation were set to 1 × 10^−6^ eV and 0.02 eV/Å, respectively.

Solvent effects were described using the implicit solvation model implemented in the VASPsol module [26], which is based on the linearized Poisson–Boltzmann (LPB) model. The relative dielectric constant of water was set to 78.4, and the surface tension parameter was fixed at 0.000525 eV/Å^2^. A Debye length of 3.0 Å was adopted to simulate the ionic screening effect of the 0.1 M KOH alkaline electrolyte. All symmetry operations were disabled throughout the calculations to avoid convergence issues arising from symmetry breaking induced by solvent polarization.

The HER in alkaline electrolyte proceeds via two well-established mechanisms: the Volmer–Heyrovsky and Volmer–Tafel pathways, both of which involve a two-electron transfer process. The initial Volmer step corresponds to water dissociation to form adsorbed hydrogen (H*) and hydroxide ions (OH^−^) [27]:

H_2_O + e^−^ + * → H* + OH^−^ (1)

Following the Volmer step, H_2_ can be generated via the Heyrovsky step:

H* + H_2_O + e^−^ → H_2_ + OH^−^ (2)

or the Tafel step:

H* + H* → H_2_ (3)

where * denotes an active adsorption site on the catalyst surface.

All Gibbs free energy calculations were performed using the computational hydrogen electrode (CHE) model [28] at 298 K and 1 bar. The reversible hydrogen electrode (RHE) was used as the reference potential scale, which inherently compensates for the pH dependence of electrochemical reactions; thus, no additional pH correction was required for any elementary step.

The Gibbs free energy change (Δ*G*) of each elementary reaction step was calculated as:

Δ*G* = Δ*E*_DFT_ + Δ*E*_ZPE_ − TΔ*S* (4)

where Δ*E*_DFT_ is the total electronic energy difference obtained from DFT calculations, Δ*E*_ZPE_ is the zero-point energy correction derived from vibrational frequency calculations, and TΔ*S* is the entropic contribution at 298 K. The entropy of gaseous H_2_ was taken from standard experimental thermodynamic data, while the entropic contributions of all surface-adsorbed intermediates were assumed to be negligible. Reaction activation barriers were determined using the CI-NEB method [29]. NEB calculations were considered converged when the maximum force on each image was lower than 0.05 eV/Å. The highest-energy structure along the optimized minimum energy pathway (MEP) was identified as the transition state (TS). TS geometries were further refined via the dimer method, and vibrational frequency analysis was performed to confirm that each TS exhibits exactly one imaginary frequency along the reaction coordinate. For electronic structure analysis, total and partial density of states (PDOS) calculations, charge density difference and Bader charge analysis were employed to elucidate electronic structure evolution and charge transfer behavior, with the corresponding Bader charge results and charge density difference map provided in the Electronic Supplementary Material (ESM).

## 3. Results and Discussion

In alkaline electrocatalytic HER, the hydrogen adsorption free energy (Δ*G*_H*_) alone is insufficient as a descriptor to fully rationalize the catalytic activity, as water dissociation is widely recognized as the rate-determining step (RDS) for the overall reaction in alkaline media. Specifically, the Volmer step, corresponding to the rate-limiting water dissociation process, involves the cleavage of the O–H bond in H_2_O to form adsorbed H* and OH* intermediates, as described by:

H_2_O + * → H* + OH* (5)

**Figure 1:**
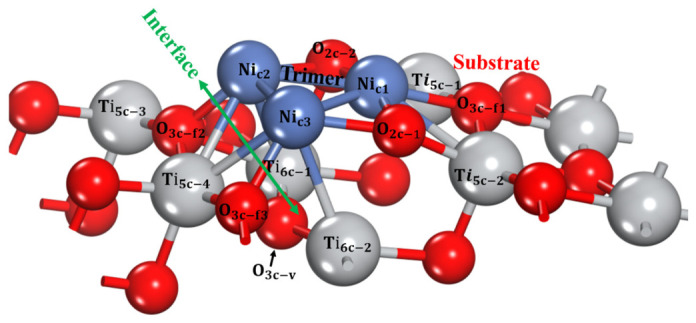
Atomic structure of the trimer Ni_3_ supported on anatase TiO_2_ (101) used in our calculations. Only the topmost Ti_2_O_4_ layer (featuring coordinatively unsaturated O_2c_ and Ti_5c_ sites, as well as coordinatively saturated Ti_6c_ and O_3c_ sites) and the supported Ni_3_ trimer are shown for clarity. Atomic subscripts refer to position numbers (e.g., Ni_c1_ is located at the center of the cavity on the (101) surface). For additional details, refer to References [21,22]. The three functionally distinct regions (substrate, trimer, and interface) are marked by a green oval, yellow triangle, and black rectangle, respectively. Blue, gray, and red spheres represent Ni, Ti, and O atoms.

Unlike the HER in acidic media [30,31], the steric hindrance associated with H_2_O adsorption and dissociation on the catalyst surface is a non-negligible factor in alkaline electrolytes. This arises from the much larger molecular size of H_2_O compared to protons, as well as the requirement for specific adsorption configurations to enable efficient O–H bond cleavage. To unravel the intrinsic activity origin of the Ni_3_/TiO_2_ catalyst, we divided the catalytic interface into three functionally distinct regions: the TiO_2_ substrate, the Ni_3_ trimer, and the Ni_3_–TiO_2_ perimeter interface (Figure 1). The individual contribution of each region to water adsorption, O–H bond activation, and intermediate desorption is systematically investigated in the subsequent sections.

**Figure 2:**
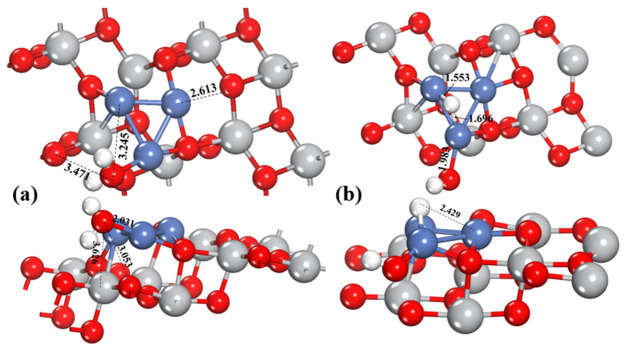
Stable adsorption configurations of (**a**) the H_2_O* molecule and (**b**) co-adsorbed OH*/H* on Ni_3_/TiO_2_ (top view in the upper panel, and side view in the lower panel). Blue, gray, red, and white spheres represent Ni, Ti, O, and H atoms, respectively.

First, for the Volmer reaction, we performed comprehensive structural optimizations and total energy calculations of the reactant (H_2_O*) and products (H* and OH*) on different regions of the Ni_3_/TiO_2_ surface, considering all high-symmetry adsorption sites (Figure S1 and all corresponding adsorption energies summarized in Table S1 in the ESM). The most stable adsorption configurations of H_2_O*, H*, and OH* are presented in Figure 2. Notably, although water is a highly polar molecule prone to polarization under electric fields, the strong coordinative interaction between water and the undercoordinated Ti sites on TiO_2_, as well as the Ni sites in the trimer, predominates over local electric field effects, giving rise to well-defined adsorption configurations that are insensitive to minor fluctuations in the local electric field. The adsorption behavior of H_2_O exhibits a pronounced site-dependent feature across the three functional regions of the Ni_3_/TiO_2_ catalyst. Specifically, adsorbed H_2_O* on the Ni sites adopts a configuration parallel to the (010) facet and perpendicular to the [100] groove, with a large adsorption energy (Figure 2a). This adsorption energy (−0.56 eV) is less than that reported for Pt_1_/TiO_2_ SACs (−0.69 eV, converted from −15.8 kcal/mol) under equivalent computational settings of DFT, but without implicit solvation model [32], and nearly identical to the water adsorption energy at the five-coordinate Ti_5c-1,2_ sites on the surface. By contrast, the adsorption energy of H_2_O on the Ni_c2,3_ atoms along the [100] groove direction decreases to −0.35 eV. Both of these values are significantly more negative more favorable than those at the Ti_5c-3,4_ sites at the open interface (−0.22 eV) and the vertically adsorbed configuration on Ni_c2_/Ni_c3_ sites (−0.23 eV). Most remarkably, H_2_O molecules are strongly repelled by the Ni_c1_ atom and cannot form stable adsorption complexes on this site.

**Figure 3:**
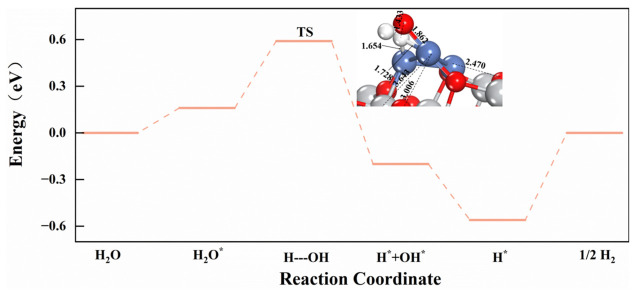
Gibbs free energy profile of the alkaline HER on Ni_3_/TiO_2_ via the Volmer–Heyrovsky reaction pathway. The atomic configuration of the transition state (TS) is shown in the inset.

Similar to the adsorption behavior of H_2_O*, the co-adsorption of H* and OH* on the Ni_3_/TiO_2_ surface also exhibits pronounced site dependence. In sharp contrast to the multiple stable adsorption configurations of H_2_O*, only one stable co-adsorption configuration was identified, with the energetically most favorable structure located exclusively at the Ni_3_–TiO_2_ interface. The adsorption energy differences between the most stable configuration and the other energetically closest metastable structures are 0.25, 0.31 and 0.54 eV, respectively. Note that for the configurational screening of OH* and H* co-adsorption structures, we referred to our previous findings from acidic HER studies, where we confirmed that hydrogen atoms preferentially adsorb on the Ni_3_ trimer rather than the TiO_2_ support [5]. Accordingly, we anchored the H* adsorbate on the Ni_3_ trimer, with initial positions set at all typical high-symmetry sites (top, bridge, and hollow sites). Using these H* adsorption configurations as starting points, we systematically screened the adsorption of OH* at all remaining high-symmetry sites. In total, 22 initial structural models were constructed, covering all available top sites, as well as Ni–O, Ni–Ti bridge sites (Figure S2 in the ESM). In this stable configuration, OH* binds to the Ni_c3_ site via its O atom, adopting an H-down orientation with the H atom pointing toward the adjacent three-coordinate O_3c-f3_ site on the TiO_2_ substrate (Figure 2b). The dissociated H* atom occupies the bridge site between Ni_c2_ and Ni_c3_, with asymmetric Ni–H bond lengths of 1.553 Å and 1.696 Å. A critical insight from our structural analysis is the exceptional geometric match between the most stable H_2_O* adsorption configuration (Figure 2a) and the energetically preferred H* + OH* co-adsorption state (Figure 2b). Specifically, the distance between the O atom of H_2_O* and the Ni_c3_ site, as well as the distance between the dissociable H atom of H_2_O* and the Ni_c2_–Ni_c3_ bridge site, are nearly perfectly matched to the corresponding distances in the product state. This distance-matching characteristic minimizes the structural rearrangement required during O–H bond cleavage, which inherently reduces the activation energy barrier. Based on the above rigorous structural analysis, these two configurations were selected as the initial state (IS) and final state (FS) for CI-NEB calculations of O–H bond cleavage in the Volmer step. The Gibbs free energy diagram for the Volmer–Heyrovsky pathway, including all adsorbed intermediates, is presented in Figure 3.

As illustrated in Figure 3, the energy barrier for H–OH bond cleavage in the Volmer step is calculated to be only 0.43 eV. This barrier is markedly lower than those reported for state-of-the-art alkaline HER catalysts, including IrRu DACs (0.68 eV) [33], bulk liquid water (intrinsic O–H bond dissociation barrier: 0.76 eV) [34], and MoS_2_/NiFe-layered double hydroxide heterostructures (0.64 eV) [35]. Such an ultralow barrier enables facile water dissociation at ambient temperature with negligible kinetic constraints. This bond dissociation process is exothermic with an energy release of 0.79 eV, which maintains the Gibbs free energy of the formed OH* + H* intermediates at a negative value (stable). Subsequently, the transformation from the OH* + H* co-adsorption state to the isolated H* state, corresponding to the reductive desorption of OH* as OH^−^ from the Ni_c3_ site, exhibits a downhill free energy decrease of 0.36 eV. This facile OH^−^ desorption effectively avoids active site poisoning by strongly adsorbed hydroxyl intermediates, thus ensuring sustainable cyclic utilization of the catalyst. The subsequent generation of H_2_ via the Heyrovsky step is an uphill process with a free energy rise of 0.56 eV. Although this value is slightly higher than the kinetic barrier for H–OH bond cleavage, both fall within a low energy range. These results not only demonstrate that Ni_3_/TiO_2_ is a promising candidate for alkaline HER, but also confirm that the overall H_2_ production proceeds smoothly along the Volmer–Heyrovsky pathway. Consequently, the RDS of the overall reaction shifts from water dissociation (the Volmer step with an activation barrier of 0.43 eV, typical for most reported alkaline HER catalysts) to the Heyrovsky step with an activation barrier of 0.56 eV. Given the negligible energy difference between the two barriers (~130 meV), either step may act as the RDS under practical electrocatalytic operating conditions. Overall, our results confirm that the Ni_3_/TiO_2_ catalyst drastically reduces the water dissociation barrier, which addresses the most long-standing and intractable bottleneck in alkaline HER.

We further elucidate the intrinsic origin of the facile H–OH bond cleavage from the perspective of TS geometry (inset of Figure 3). The two Ni atoms on the Ni_c2_–Ni_c3_ edge at the interfacial step are coordinated with two-coordinate (O_2c_) and three-coordinate (O_3c_) lattice oxygen atoms, respectively. With only moderate electron transfer (0.34 e for Ni_c2_ and 0.39 e for Ni_c3_), these Ni sites retain high metallic character and strong hydrogen affinity, analogous to bulk metallic Ni [28]. Consequently, this edge region exerts a strong attractive interaction on the dissociable hydrogen atom of the adsorbed H_2_O molecule, with H–Ni_c2_ and H–Ni_c3_ distances of 1.654 Å and 1.728 Å, respectively. These distances are slightly longer than the corresponding values for the bridge-adsorbed H* in the FS. At the TS, the distance between the dissociating H atom and the O atom of the residual OH group on Ni_c3_ reaches 1.433 Å, which is considerably longer than the typical O–H bond length of 0.96 Å in free H_2_O. This extended bond length (nearly half the intrinsic O–H bond distance) strongly indicates the occurrence of O–H bond scission, which is further confirmed by a single imaginary frequency of 622.65 *i* cm^−1^ along the reaction coordinate. The residual OH* species remains adsorbed on the Ni_c3_ site with an orientation perpendicular to the edge, which is similar to that in the IS, albeit with a slight upward shift. Meanwhile, the O–Ni_c3_ bond length decreases from 2.031 Å (IS) to 1.862 Å (TS).

Intriguingly, Bader charge analysis (Table S2 in the ESM) provides further electronic-level insight into the favorable reaction behavior. At the FS, the OH* intermediate accepts 0.76 e from the Ni_3_ trimer, exhibiting a pronounced hydroxylation tendency. This electronic redistribution is in excellent agreement with the low desorption free energy of OH* observed in our thermodynamic calculations. Meanwhile, the dissociated H atom anchored at the Ni_c2_–Ni_c3_ bridge site exists in a nearly neutral atomic state. Notably, the adjacent Ni_c2_ atom bound to the dissociated H* undergoes the least electron loss of only 0.16 e. In sharp contrast, Ni_c1_, which has no direct interaction with the intermediates, exhibits an identical electron loss of 0.23 e as Ni_c3_. The majority of the electrons donated from the Ni_3_ trimer are transferred to the adsorbed OH* species, which directly confirms the delocalized electronic nature of the Ni_3_ trimer. Under electrocatalytic conditions, a net current from the external circuit flows during hydrogen evolution at the equilibrium potential (U = 0 V vs. RHE) [28], leading to significant electron accumulation on the cathodic Ni_3_ trimer, independent of the substrate. The electron-enriched Ni_3_ trimer actively attracts polarized water molecules via electrostatic interactions and further promotes their polarization, drawing the H species of H_2_O (effectively H^+^) toward the cathode. Once one electron (e^−^) is transferred to the adjacent H^+^, the latter is reduced to a H atom, triggering O–H bond cleavage of H_2_O and chemisorption of the H atom onto the trimer. These discussions are fully validated by our aforementioned NEB calculations and Bader charge analysis results. In particular, the repulsive interaction between the electron-rich Ni_3_/TiO_2_ cathode and the hydroxylated OH^−^ group inevitably drives OH^−^ desorption from the catalyst surface into the bulk media. The charge states of individual Ni atoms on the H-adsorbed trimer tend to be uniform, with a total charge transfer of 0.54 e compared with the clean surface without H adsorption (Table S2 in the ESM).

This indicates that once the reduction event occurs, the adsorption behavior of H* on Ni_3_/TiO_2_ is insensitive to the pH of the electrolyte (acidic or alkaline conditions). In this context, Ni_3_/TiO_2_ can still provide available active sites for the dissociation of a second water molecule, which is involved in H_2_ generation via Equation (2) and the retention of the second adsorbed H* via Equation (3), following either the Volmer–Heyrovsky or Volmer–Tafel mechanism. Different from SACs and DACs, a unique advantage of TACs is that the Ni_3_ trimer can provide abundant active sites to simultaneously accommodate fresh reactants and reaction intermediates, reflecting the inherent structural diversity and flexibility of TACs. This feature enables TACs to be compatible with more types of chemical reactions and fundamentally improve the selectivity of multi-step reactions. However, as an active moiety of the electrode (in contrast to the inert substrate), the Ni_3_ trimer has a limited surface area and spatial extent. This leads to the accumulation of reactants and intermediates on the Ni_3_ moiety during the reaction (typically at different sites, accompanied by structural flexibility), resulting in inevitable interactions between adsorbed species which can be either favorable or unfavorable for the HER rates. This behavior is distinctly different from the HER on bulk metal surfaces, where individual reaction steps and adsorption processes occur independently at separate sites, and adsorbed species only diffuse together for the final H_2_ formation.

**Figure 4:**
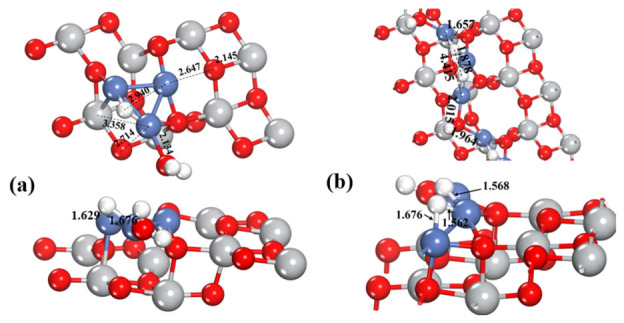
Stable adsorption configurations of (**a**) the H_2_O* molecule and (**b**) co-adsorbed OH*/H* on H-Ni_3_/TiO_2_ (top view in the upper panel, and side view in the lower panel). Blue, gray, red, and white spheres represent Ni, Ti, O, and H atoms, respectively.

**Figure 5:**
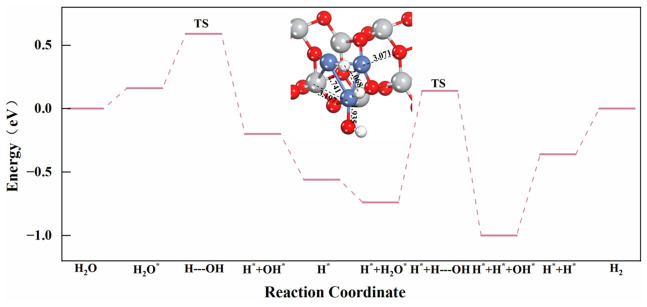
Gibbs free energy profile of the alkaline HER on Ni_3_/TiO_2_ via the Volmer–Tafel reaction pathway. The atomic configuration of TS, with a single imaginary frequency of 166.81 *i* cm^−1^ along the reaction coordinate, is shown in the inset.

Hereinafter, Ni_3_/TiO_2_ with a single pre-adsorbed H* atom is denoted as H-Ni_3_/TiO_2_. Within the framework of DFT and CI-NEB calculations, we obtained the stable adsorption configuration of H_2_O on H-Ni_3_/TiO_2_, with a corresponding adsorption energy of −0.76 eV. The adsorbed H_2_O* binds to the Ni_c3_ site and aligns along the [100] groove (Figure 4a), which is distinct from the water activation behavior and the contribution of oxide supports in SACs under alkaline or acidic conditions [21,32]. This difference arises from the Coulombic repulsion between the adsorbed H_2_O* and the pre-adsorbed H* on the bridge site of Ni_c2_–Ni_c3_ edge. To clarify this repulsive interaction, a symmetric isosurface value of ±0.003 e/Å^3^ was adopted to plot the differential charge density map for the co-adsorption of H* and H_2_O* on Ni_3_/TiO_2_ (Figure S3 in the ESM). The yellow isosurface denotes electron accumulation, and the cyan isosurface represents electron depletion. According to the charge distribution, both the Ni_3_ trimer and adsorbed H* atom exhibit electron accumulation, while the adsorbed H_2_O* molecule shows electron depletion, accompanied by a clear boundary between electron gain and loss in the intermediate region. This observation is highly consistent with the Bader charge analysis results (Table S2 in the ESM). Notably, an evident cyan negative isosurface emerges between the adsorbed H* atom and the O atom of the water molecule, indicating the formation of an electron depletion region. This region completely separates the electron clouds of the H* atom and H_2_O* molecule, suggesting that electrons are repelled away from both species. This behavior is a typical feature of Coulomb repulsion among like-charged electrons. To further corroborate this hypothesis, we selected the optimized configuration H_2_O* as the IS, and the configuration with H_2_ parallel to the trimer at a distance of ~4.01 Å as the FS for CI-NEB calculations. During the calculations, we identified a stable intermediate state (designated as FS1, see Figure 4b) associated with the second water dissociation event. In the FS1, the dissociated OH* is confined within the [100] groove and bridges Ni_c2_ and Ni_c3_ sites, with Ni–O bond lengths of 2.015 Å and 1.964 Å, respectively. This adsorption is accompanied by the cleavage of the original Ni_c2_–Ni_c3_ metallic bond, with its bond length increasing from 2.344 Å to 4.415 Å. Such bond breakage is attributed to the stress concentration and electronic state saturation of the Ni_3_ trimer, induced by the accumulation of multiple reaction intermediates in the confined space of the trimer, independent of the TiO_2_ substrate. The physical origin of this behavior is elucidated in detail as follows. The dissociation of the second water molecule proceeds predominantly on the Ni_3_ trimer. Bader charge analysis reveals that the dissociated OH* intermediate accepts 0.62 e from the Ni_c2_ and Ni_c3_ atoms. Such intense charge transfer significantly reduces the electron density between Ni_c2_ and Ni_c3_, thereby weakening the metallic bonding interaction between these two atoms. In particular, the Ni_c3_ site directly bonded to the OH* group exhibits the lowest charge population, making it difficult for the pre-adsorbed H* atom to extract electrons from Ni_c3_ and maintain its atomic adsorbed state. Meanwhile, constrained by spatial steric hindrance, the newly dissociated H* atom can only be trapped at the bridge site near the Ni_c1_–Ni_c3_ edge. The mutual electrostatic repulsion between the two adsorbed H* atoms first weakens and eventually breaks the H*–Ni_c3_ bond. Subsequently, the H* atom rotates to bond with Ni_c1_ and occupies the bridge site at the Ni_c1_–Ni_c2_ edge. Compared with the free Ni_3_ trimer, the Ni_3_ trimer supported on the TiO_2_ (101) surface displays elongated interatomic bond lengths, indicating that the trimer is subjected to tensile stress imposed by the TiO_2_ substrate. During the dissociation of the second water molecule, the combined effects of electron transfer and spatial confinement further amplify this tensile stress, as evidenced by the pronounced bond length variations between the coordinated and central Ni atoms. The intensified stress is primarily localized on the already weakened Ni_c2_–Ni_c3_ bond. The free migration of Ni_c2_ along the surface groove represents the optimal pathway for stress relaxation without spatial obstruction. Furthermore, Ni_c2_ is attracted by the adsorbed OH* species on Ni_c3_, which induces partial electron backflow toward Ni_c2_ to compensate its valence state. This is consistent with the higher charge population of Ni_c2_ relative to Ni_c3_ (Table S2 in the ESM). Ultimately, the OH* group bridges the two Ni active sites and triggers the cleavage of the Ni_c2_–Ni_c3_ bond (see the transition state structure in Figure 5). In summary, the rupture of the Ni_c2_–Ni_c3_ bond is an inevitable structural evolution during the second water dissociation process. The electron redistribution and structural distortion originating from steric hindrance elevate the reaction activation barrier, whereas the release of concentrated stress facilitates barrier reduction. This well explains why, although the Ni_3_ trimer provides abundant active sites to promote the dissociation of the second water molecule, its corresponding energy barrier is considerably higher than that for the first water dissociation (i.e., the Volmer step). This phenomenon has been reported in previous studies [8], but is absent in SACs or supported nanoparticle catalysts. This structural flexibility gives rise to two critical and unexpected findings. First, the Tafel pathway, which requires two adsorbed H* species, involves two distinct water dissociation steps on Ni_3_/TiO_2_ with significantly different dissociation barriers. Second, stress-driven structural deformation enables a more flexible evolution of the reaction barrier along the catalytic cycle, through the release or absorption of energy and distance-matching between reactants and products. The Gibbs free energy diagram for the Volmer–Tafel reaction pathway, including all adsorbed intermediates, is presented in Figure 5.

It is noteworthy that the first half of Figure 5 largely overlaps with the core portion of Figure 3. The process from H_2_O* adsorption to dissociation generates an adsorbed H* species stabilized on the Ni_3_ trimer. This pathway not only recapitulates the aforementioned Volmer–Heyrovsky mechanism but also supplies the first adsorbed H* prerequisite for the Tafel reaction. Proceeding along the reaction coordinate in Figure 5, a second H_2_O dissociation event is required to produce a second adsorbed H* on the Ni_3_ trimer, which is indispensable for triggering the Tafel reaction. Importantly, the Ni_3_/TiO_2_ catalyst features sufficient active sites to accommodate this sequential two-step water dissociation, as illustrated in the latter half of Figure 5.

Notably, the pre-adsorbed H* species promotes the adsorption of the second H_2_O molecule, which proceeds with a downhill free energy change of 0.18 eV. Subsequently, on the H-Ni_3_/TiO_2_ surface, the second water dissociation step (required to generate the second H* for the H* + H* → H_2_ recombination) has an energy barrier of 0.88 eV, ~2.0 times higher than that of the first Volmer step (0.43 eV). This step is highly exothermic, releasing 1.14 eV of energy, which is significantly larger than the 0.79 eV released in the first Volmer step. Intriguingly, the charge states of the H* and OH* species generated from the second water dissociation are nearly identical to those from the first dissociation event. Specifically, the dissociated H* remains in a nearly neutral atomic state, while the OH* intermediate accepts 0.62 e^−^ from the catalyst system and exhibits characteristic anionic character. Driven by strong electrostatic repulsion from the electron-enriched Ni_3_/TiO_2_ surface, the anionic OH* species readily detaches from the catalytic interface. Concomitantly, the accumulated lattice strain within the Ni_3_ trimer is fully relieved, as evidenced by the contraction of the Ni_c2_–Ni_c3_ bond length to 2.467 Å from its initial value. This structural recovery enables the reformation of the Ni_c2_–Ni_c3_ metallic bond, restoring the intact triangular configuration of the Ni_3_ trimer. Following OH* desorption, a dihydride species forms on the Ni_c1_ site, accompanied by the concerted migration of two edge-bound H* species—located at the bridge sites of the Ni_c1_–Ni_c2_ and Ni_c1_–Ni_c3_ edges, with an H*–H* separation of 2.116 Å and a Ni_c2_–Ni_c3_ bond length of 3.227 Å—to the Ni_c1_ active center. Collectively, these two processes are endothermic by a total of 0.64 eV, in sharp contrast to the single OH* desorption step along the Volmer–Heyrovsky pathway, which is exothermic by 0.36 eV. Finally, the recombination of two adsorbed H* to form H_2_ (the Tafel step, corresponding to chemical desorption) is an endothermic process with an energy barrier of 0.36 eV, which is lower than the 0.56 eV barrier of the Heyrovsky step (electrochemical desorption). Upon the final formation and desorption of H_2_, the Ni_3_/TiO_2_ catalyst fully reverts to its initial pristine state prior to the catalytic cycle, owing to the high structural stability of the Ni_3_ trimer anchored on the TiO_2_ (101) surface [3,5,21,22], thus completing the entire alkaline HER process via the Volmer–Tafel pathway. Taken together, despite the presence of multiple active sites on Ni_3_/TiO_2_, the requirement for a second water dissociation to generate the additional H* renders the Volmer–Tafel pathway kinetically less favorable than the Volmer–Heyrovsky pathway. The intrinsic origin of this distinct kinetic behavior can be further elucidated by analyzing the electronic structure evolution of the Ni_3_ trimer and its orbital interactions with reaction intermediates.

**Figure 6:**
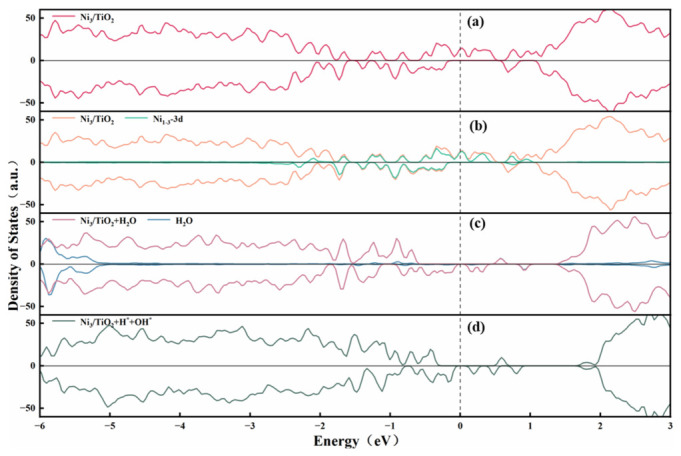
Total and partial density of states (TDOS and PDOS) of the alkaline HER on Ni_3_/TiO_2_ via the Volmer–Heyrovsky reaction pathway. (**a**) TDOS for the Ni_3_/TiO_2_ system; (**b**) TDOS for the Ni_3_/TiO_2_ system (orange) and PDOS for Ni_3_-d (green); (**c**) TDOS for the Ni_3_/TiO_2_ + H_2_O system (crimson) and PDOS for H_2_O (dark green); (**d**) TDOS for the Ni_3_/TiO_2_ + H* + OH* system (grass green). The Fermi level (E*_F_*) is set to 0 eV.

**Figure 7:**
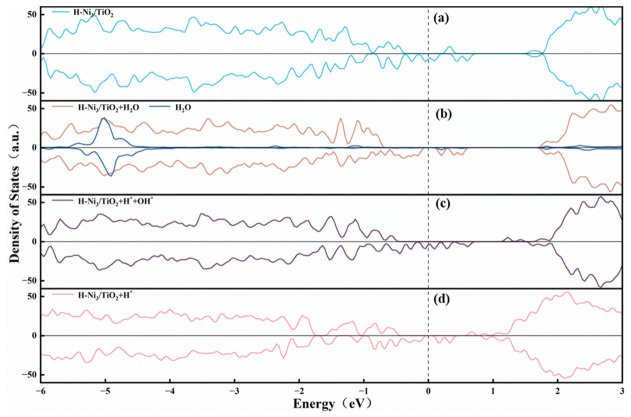
TDOS and PDOS of the alkaline HER on Ni_3_/TiO_2_ via the Volmer–Tafel reaction pathway. (**a**) TDOS for the H-Ni_3_/TiO_2_ system; (**b**) TDOS for the H-Ni_3_/TiO_2_ + H_2_O system (brown) and PDOS for H_2_O (dark blue); (**c**) TDOS for the H-Ni_3_/TiO_2_ + H* + OH* system; (**d**) TDOS for the H-Ni_3_/TiO_2_ + H* system. The Fermi level (E*_F_*) is set to 0 eV.

We address the quantum confinement effect of the Ni_3_ trimer by analyzing spatial localization/delocalization of metallic states of Ni_3_/TiO_2_ on the alkaline HER, following the Volmer–Heyrovsky and Volmer–Tafel pathways. The total and partial density of states (TDOS and PDOS) of intermediates at each stage of the hydrogen production process are summarized in Figure 6 and Figure 7.

Figure 6a,b reveal that the continuous and extended metallic state of the Ni_3_ trimer fully fills the band gap of anatase TiO_2_. This is mainly attributed to the hybridization between Ni-3d orbitals and coordinated O-2p orbitals at the valence band maximum (VBM), which stabilizes the immobilization of the Ni_3_ trimer on the TiO_2_ substrate. Meanwhile, Bader charge analysis confirms a total of 1.24 e is transferred from the Ni_3_ trimer to the TiO_2_ support. Since the total electron number of the reactant H_2_O molecule and the product OH*/H* species is far smaller than that of the Ni_3_/TiO_2_ catalyst, their contributions to the overall DOS are negligible in the plots. Therefore, we tracked the evolution of the electronic states of the Ni_3_ trimer before and after water dissociation to unravel the intrinsic origin of the reaction barrier.

As shown in Figure 6c, the adsorption of H_2_O molecule leads to the electronic states of the Ni_3_ trimer being more concentrated below the E*_F_*. Meanwhile, the polarization effect of the adsorbed H_2_O induces a more pronounced polarization splitting of the metallic states, resulting in the absence of electronic states at the E*_F_*. Notably, this weak adsorbate–catalyst interaction does not alter the total amount of electron transfer from the Ni_3_ trimer to the TiO_2_ substrate; instead, it only triggers charge redistribution among the three Ni atoms via the Ni–Ni metallic bonds. In contrast, Figure 6d shows that the co-adsorption of H* and OH* intermediates still maintains the metallic states of the Ni_3_ trimer mainly below the E*_F_*, but with a more uniform distribution. The polarization splitting is significantly weakened, and the overall electronic states shift closer to the E*_F_*. Figure 6c,d further reveal that the number of electronic states in the unoccupied states is far lower than that in the occupied states, which visually demonstrates that the electronic states of the Ni atoms are saturated, thus endowing the Ni_3_ trimer with higher structural stability. Throughout the entire water dissociation process, the metallic states of the Ni_3_ trimer consistently maintain a pronounced continuous and extended character, indicating that the crystal embryo-like Ni_3_ trimer possesses an embryonic metallic nature. This is consistent with the results inferred from its lattice orientation behavior as reported in reference [5]. The driving force for the O–H bond cleavage originates from the intrinsic requirement of the transition of the metallic electronic states from a localized to a delocalized character, while the attenuation of polarization splitting is only a concomitant result of this electronic transition. This result fully confirms the confinement effect of delocalized metallic states among TM atoms.

For the H-Ni_3_/TiO_2_ system, the desorption of OH* reduces the polarization effect, leading to the emergence of a small number of electronic states at the E*_F_* (Figure 7a), accompanied by a further upward shift in the d-band center. Upon the re-adsorption of a second water molecule, a consistent trend is observed: the electronic states shift toward deeper energy levels, with a concomitant downward shift in the d-band center. However, compared with Figure 6c, the overall downward shift in the electronic states in Figure 7b is less pronounced in the energy range of −0.4 eV to −1.5 eV, and the spin splitting near the E*_F_* is more distinct. This result directly reflects a stronger binding interaction between the water molecule and the H-Ni_3_/TiO_2_ surface. Since the pre-adsorbed H* has already occupied the optimal product site for water dissociation, the newly generated H* from the second water dissociation must migrate to a new favorable adsorption site, such as the Ni_c1_–Ni_c3_ edge. This process forces the original pre-adsorbed H* to leave its optimal site and migrate toward the Ni_c1_–Ni_c2_ edge. Such structural rearrangement induces the cleavage of the Ni_c2_–Ni_c3_ bond, with the concentrated structural stress evolving synchronously with the structural deformation. On the other hand, the two dissociated H* species adopt equivalent adsorption configurations, as evidenced by Figure 7c. The spin splitting of the metallic states is significantly reduced, and the electronic states are mainly concentrated near the E*_F_*, which is dominated by the contribution from the adsorbed OH* intermediate. Notably, no obvious spin splitting is observed for energy levels below −0.4 eV, where the electronic states exhibit a uniform and homogeneous distribution. Meanwhile, the d-band center shifts upward, corresponding to the enhanced electronic delocalization of the Ni_3_ trimer. Figure 7d shows that after OH* desorption, the non-equivalent occupation of adsorption sites by the two H* species, combined with the residual structural deformation, leads to a slight increase in spin splitting and a less homogeneous distribution of the electronic states.

In short, our calculations indicate that the hydrogen production process over the Ni_3_/TiO_2_ TAC in alkaline media is dominated by the Volmer–Heyrovsky reaction, with the RDS being the Volmer or Heyrovsky step. To the best of our knowledge, this unique mechanistic feature has not been previously reported or systematically investigated in studies on HER over FACs. It is unclear whether this phenomenon was unmentioned or intentionally overlooked, even in related studies on SAC/DAC-based HER systems. For the Ni_3_/TiO_2_ TAC, the HER exhibits a low kinetic barrier that is theoretically favorable for alkaline HER at ambient temperature. Accordingly, its theoretical alkaline HER activity is comparable to that of state-of-the-art non-noble metal acidic HER catalysts under the same computational framework [33,34,35].

In alkaline HER, the water dissociation barrier, which typically acts as the RDS, is widely recognized as the primary descriptor for evaluating catalytic activity. Accordingly, Table 1 summarizes the Volmer reaction barrier values of the Ni_3_/TiO_2_ catalyst system and several state-of-the-art alkaline HER catalysts reported recently. As consistently established earlier, a lower RDS energy barrier correlates with enhanced catalytic activity and facile reaction kinetics at room temperature. Strikingly, for the Ni_3_/TiO_2_ catalyst, the activation barrier of the Volmer step is markedly lowered, rendering it comparable to that of the subsequent Heyrovsky electrochemical hydrogen desorption step. In fact, both activation barriers are maintained at ~0.5 eV, indicating that the alkaline HER process over Ni_3_/TiO_2_ proceeds spontaneously at room temperature without appreciable kinetic constraints. As a direct consequence, the long-standing bottleneck of intrinsically sluggish water dissociation kinetics that limits overall HER efficiency is effectively overcome for the Ni_3_/TiO_2_ catalyst. Most importantly, these findings revise the long-held consensus that water dissociation is the sole RDS for alkaline HER, clearly demonstrating that the Heyrovsky reaction can instead become the dominant RDS under optimized conditions. Beyond revising the fundamental mechanistic understanding, in addition to providing abundant active sites for the Volmer–Heyrovsky or Volmer–Tafel pathway, the Ni_3_/TiO_2_ catalyst exhibits a lower overall reaction barrier than most reported SACs and DACs for alkaline HER (Table 1).

Collectively, the present findings carry substantial practical and theoretical implications. Building upon these fundamental mechanistic insights, we propose a rational design strategy: enhancing the intrinsic activity of edge-interfacial sites in few-atom catalysts (FACs) to lower the overall reaction energy barriers. Crucially, this approach affords superior catalytic performance compared to state-of-the-art SACs and DACs reported in the latest literature, thereby addressing the persistent challenge of sluggish reaction kinetics in alkaline hydrogen production.

Fundamentally, our combined Bader charge analysis and PDOS calculations unambiguously demonstrate that the Ni_3_ trimer possesses pronounced electronic delocalization that undergoes dynamic evolution throughout the entire reaction coordinate. Specifically, these delocalized electronic states enable highly efficient charge transfer between Ni atoms and reaction intermediates. More precisely, the electron-deficient Ni_c3_ site bound to OH* can obtain efficient charge compensation from adjacent Ni atoms via the delocalized electronic network. This charge redistribution, in turn, effectively stabilizes the adsorbed H species at the bridge sites between Ni_c1_ and Ni_c2_—a critical factor underlying the exceptional alkaline HER activity of Ni_3_/TiO_2_. Taken together, these electronic structure insights, when integrated with our aforementioned mechanistic analysis, establish a comprehensive mechanistic framework for understanding the origin of the enhanced catalytic performance of Ni_3_/TiO_2_. Importantly, although direct in situ experimental visualization of this dynamic electronic delocalization remains technically challenging, our PDOS calculations unambiguously reveal that the d-band center of the Ni_3_ trimer shifts significantly toward the Fermi level relative to the Ni_1_/TiO_2_ SAC and Ni_2_/TiO_2_ DAC [5]. This theoretical prediction is in excellent quantitative agreement with the experimentally measured HER overpotential reduction, thereby providing indirect yet compelling experimental validation of the promotional role of electronic delocalization in boosting catalytic activity. By contrast, SACs and/or DACs exhibit highly localized electronic states and lack such interatomic charge transfer pathways. Consequently, the Ni_3_ trimer features significantly enhanced electronic delocalization and constructs more robust and efficient charge transfer channels, which constitute the fundamental electronic origin of the superior HER performance of this triatomic catalyst relative to its SA and DA analogs.

## 4. Conclusions

Our calculations reveal that this low-cost non-noble-metal Ni_3_/TiO_2_ TAC, featuring delocalized electronic states confined within the trimeric Ni_3_ motif, provides a promising alternative to noble-metal-based SACs for alkaline HER. All critical reaction steps of the alkaline HER occur predominantly on the supported Ni_3_ trimers, which serve as electron-enriched active centers. This unique property not only promotes water dissociation by accelerating O–H bond cleavage and proton reduction, but also facilitates the desorption of adsorbed hydroxyl (OH*) intermediates to regenerate active sites, thus enabling sustained catalytic turnover. Mechanistically, the HER on Ni_3_/TiO_2_ TAC proceeds predominantly via the Volmer–Heyrovsky pathway. Notably, water dissociation is not the sole RDS, as the electrochemical H_2_ desorption step exhibits a nearly identical energy barrier with a difference in only 0.13 eV between the two steps. This indicates that either step can act as the RDS under varying practical electrocatalytic conditions. In contrast, the Volmer–Tafel pathway is both thermodynamically and kinetically unfavorable. This is attributed to the fact that the secondary water dissociation required to generate a second adsorbed hydrogen (H*) intermediate is severely constrained by the limited spatial extent and active site availability of the sub-nanoscale trimer, along with the steric repulsion between co-adsorbed intermediates. This reaction pathway selectivity is markedly distinct from that observed for HER in acidic media. This work establishes a viable strategy to replace noble-metal HER catalysts with non-noble metal alternatives by engineering tunable delocalized electronic states in trimeric structures to precisely regulate the adsorption and desorption behaviors of reactants, key intermediates, and products. The proposed design principle for constructing FACs to optimize catalytic activity and tune reaction mechanism is broadly applicable to complex multi-step reactions that rely on synergistic multiple active sites and the stabilization of diverse reaction intermediates.

## Figures and Tables

**Table 1:** Comparison of the kinetic barrier of water dissociation for Ni_3_/TiO_2_ catalyst system and several representative benchmark alkaline HER catalysts reported (in Unit of eV).

Catalyst	Ni_3_/TiO_2_	Pt_1_Fe_1_-DAC	Pt_1_Fe_1_-PDAC	Pt/Fe-DAC	CrCr-DAC	IrRu DAC	Co/NG SAC	Pt/TiO_2_ SAC
Kinetic barrier	0.49 (1 H_2_O); 0.88 (2 H_2_O)	0.75	2.37	2.99	2.48	0.68	1.26	1.46
Ref.	This work	[36]	[36]	[36]	[37]	[33]	[38]	[32]

## Data Availability

The original contributions presented in this study are included in the article/Supplementary Materials. Further inquiries can be directed to the corresponding author.

## Supplementary Materials

The following supporting information can be downloaded at: https://www.mdpi.com/article/10.3390/ma19112217/s1, Figure S1: All possible adsorption sites for H_2_O on the Ni_3_/TiO_2_ system (on the right). Bridge and hollow sites are described in detail (on the left). Blue, gray and red balls represent Ni, Ti and O atoms, respectively; Figure S2: All possible adsorption sites for OH* on the Ni_3_/TiO_2_ system, with the H* on the trimer. T and B indicate top and bridge sites. Blue, gray, red and white balls represent Ni, Ti, O and H atoms, respectively; Figure S3: Differential charge density map for the co-adsorption of H* and H_2_O* on Ni_3_/TiO_2_ system (top view on the left panel, and side view on the right panel). Yellow (light gray) and cyan (dark gray) isosurfaces represent the charge accumulation and depletion, respectively. The isosurface value is ±0.003 e/Å^3^; Table S1: Final adsorption energy (*E*_ad_) of H_2_O on Ni_3_/TiO_2_ after structural optimization (in Unit of eV); Table S2: Bader charge q of the central Ni atoms, surrounding coordination O atoms, adsorbed H* and OH* species on Ni_3_/TiO_2_ during two water dissociation, with electrically neutral OH and H_2_O as the reference (in Unit of e).
