# Supplementary material for: Theoretical Investigation of Hydrogen Production from Alkaline Media Through TiO2-Supported Triple-Atom Catalysts
Source: Materials (Basel). 2026 May 25;19(11):2217. doi: 10.3390/ma19112217 (PMC13258554; doi:10.3390/ma19112217)
Supplement: Supplementary file 1 [file materials-19-02217-s001.zip › materials-4311481-supplementary.pdf]

Electronic Supplementary Material

Theoretical Investigation of Hydrogen Production from Alkaline Solution through TiO_2-supported Triple-Atom Catalysts

Guangce Zhao, Gang Zhou*

School of Science, Hubei University of Technology, Wuhan 430068, People’s Republic of China

E-mail: 995372896@qq.com

Top:

T_1 T_2 T_3 T_4 T_5
T_6 T_7 T_8 T_9 T_{10}
T_{11} T_{12} T_{13} T_{14}

Ni-Ni Bridge:

B_{12} : T_1- T_2
B_{13} : T_1- T_3
B_{23} : T_2- T_3

Hollow:

H_q : H_1-H_2-H_3

Ni-O Bridge:

B_{19} : T_1-T_9
B_{114} : T_1-T_{14}
B_{113} : T_1-T_{13}
B_{29} : T_2-T_9
B_{210} : T_2-T_{10}
B_{312} : T_3-T_{12}
B_{313} : T_3-T_{13}

Ni-Ti Bridge:

B_{14} : T_1-T_4
B_{17} : T_1-T_7
B_{26} : T_2-T_6
B_{36} : T_3-T_6
B_{17} : T_1-T_7

Ti-O Bridge:

B_{49} : T_4- T_9
B_{414} : T_4- T_{14}
B_{510} : T_5- T_{10}
B_{610} : T_6-T_{10}
B_{611} : T_6-T_{11}
B_{612} : T_6-T_{12}
B_{713} : T_7-T_{13}
B_{714} : T_7-T_{14}
B_{814} : T_8-T_{14}

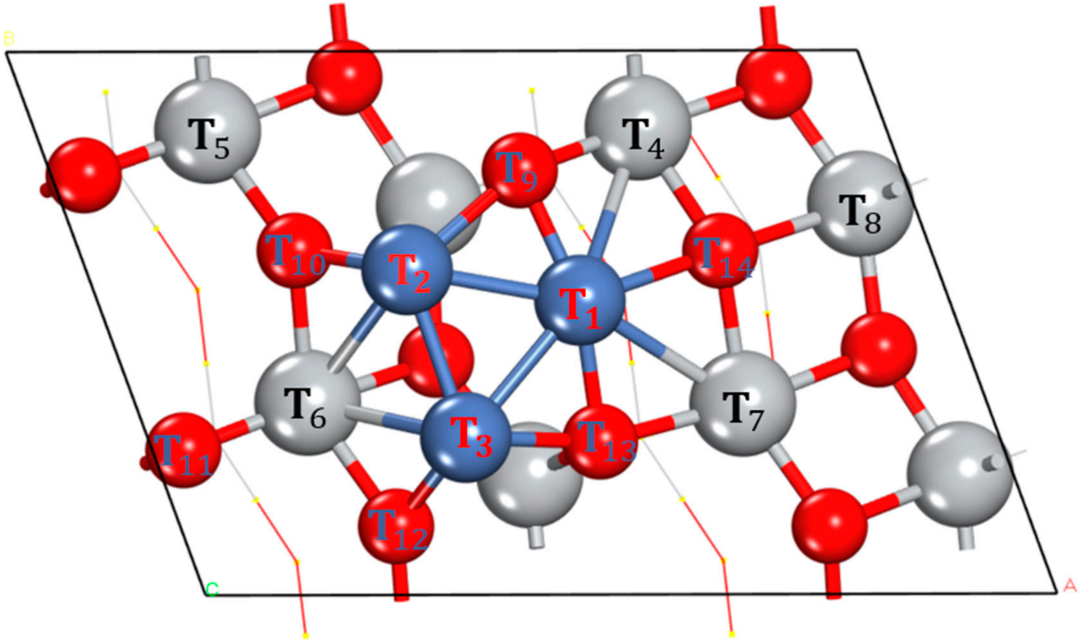

Figure S1. All possible adsorption sites for H_2O on the Ni_3/TiO_2 system (on the right). Bridge and hollow sites are described in detail (on the left). Blue, gray and red balls represent Ni, Ti and O atoms, respectively.

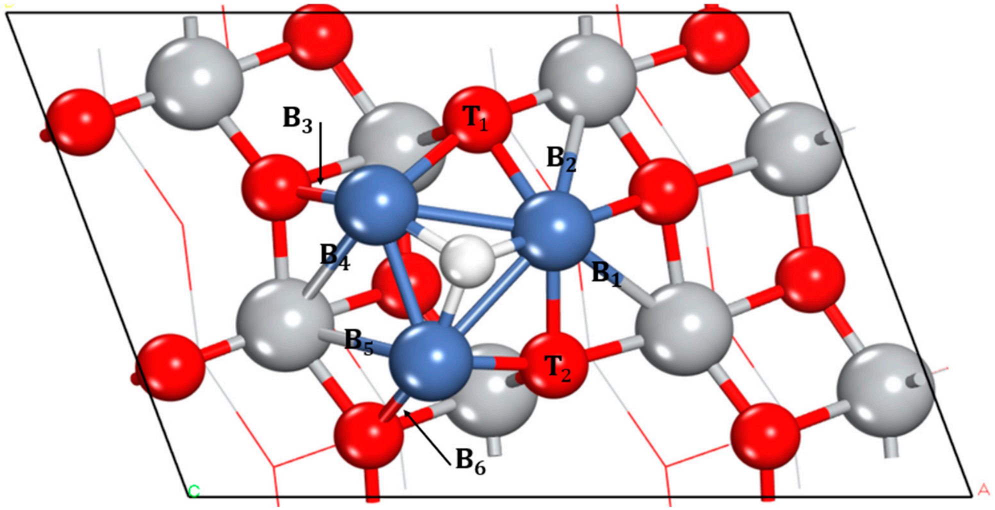

Figure S2. All possible adsorption sites for OH* on the Ni_3/TiO_2 system, with the H* on the trimer. T and B indicate top and bridge sites. Blue, gray, red and white balls represent Ni, Ti, O and H atoms, respectively.

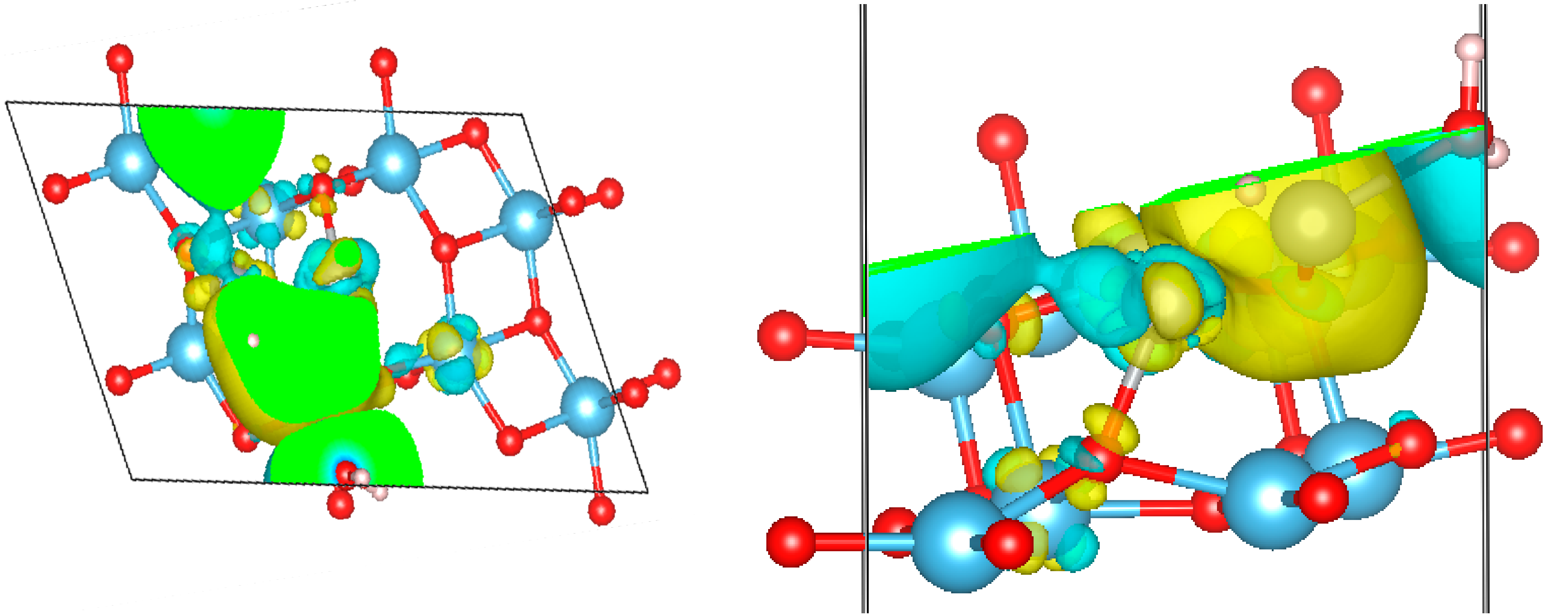

Figure S3. Differential charge density map for the co-adsorption of H* and H_2O* on Ni_3/TiO_2 system (top view on the left panel, and side view on the right panel). Yellow (light gray) and cyan (dark gray) isosurfaces represent the charge accumulation and depletion, respectively. The isosurface value is ±0.003 e/$Å^3$.

Table S1 Final adsorption energy (E_{ad}) of H_2O on Ni_3/TiO_2 after structural optimization (in Unit of eV).

Initial adsorption sites	T_1	T_2	T_3	T_4	T_5	T_6	T_7	T_8	T_9	B_{23}	H_q
E_{ad}	0.10	-0.23	-0.22	-0.56	-0.22	-0.56	-0.53	-0.11	-0.28	-0.35	-0.02

*After structural optimization, the initial adsorption configurations of the adsorption sites presented in Figure S1 evolve into the adsorption structure at the same site. Therefore, although a total of 39 initial sites were screened, only 11 final stable and metastable structures were obtained.

Table S2 Bader charge q of the central Ni atoms, surrounding coordination O atoms, adsorbed H* and OH* species on Ni_3/TiO_2 during two water dissociation, with electrically neutral OH and H_2O as the reference (in Unit of e).

	O_{2c-1}	O_{2c-2}	O_{3c-1}	O_{3c-2}	Ni_{c1}	Ni_{c2}	Ni_{c3}	H_{ad1}*	H_{ad2}*	O(OH*)	H(OH*)	O(H_2O)	H_{-1}(H_2O)	H_{-2}(H_2O)
H_2O												7.15	0.41	0.44
OH										6.56	0.44			
Ni_3/TiO_2	7.20	7.21	7.30	7.30	9.49	9.66	9.61							
Ni_3/TiO_2+H_2O	7.19	7.20	7.28	7.30	9.49	9.82	9.49					7.21	0.33	0.38
Ni_3/TiO_2+H+OH	7.14	7.16	7.30	7.26	9.26	9.66	9.26	1.25		7.24	0.38			
Ni_3/TiO_2+H (H-)	7.17	7.17	7.30	7.29	9.38	9.41	9.43	1.28						
H-Ni_3/TiO_2+H_2O	7.22	7.21	7.29	7.29	9.40	9.70	9.42	1.30				7.27	0.31	0.37
H-Ni_3/TiO_2+H+OH	7.21	7.21	7.29	7.29	9.40	9.26	9.19	1.21	1.23	7.26	0.36			
H-Ni_3/TiO_2+H	7.21	7.21	7.32	7.30	9.40	9.47	9.45	1.22	1.23					
